# Emergency Department Vestibular Rehabilitation Therapy for Dizziness and Vertigo: A Nonrandomized Clinical Trial

**DOI:** 10.1001/jamanetworkopen.2024.59567

**Published:** 2025-02-14

**Authors:** Howard S. Kim, Jacob M. Schauer, Ann K. Kan, Joshua B. Alinger, Kyle J. Strickland, Alexander Garreau, Danielle M. McCarthy, Zachary B. Taylor, Ivy L. Fishman, Kayla M. Muschong, Heidi R. Roth

**Affiliations:** 1 Department of Emergency Medicine, Northwestern University Feinberg School of Medicine, Chicago, Illinois; 2 Deputy Editor, *JAMA Network Open*; 3 Division of Biostatistics, Department of Preventive Medicine, Northwestern University Feinberg School of Medicine, Chicago, Illinois; 4 Department of Rehabilitation Services, Northwestern Memorial Hospital, Chicago, Illinois; 5 Department of Physical Therapy and Human Movement Sciences, Northwestern University Feinberg School of Medicine, Chicago, Illinois

## Abstract

**Importance:**

Dizziness symptoms account for nearly 2 million annual emergency department (ED) visits and present a diagnostic challenge for clinicians. Most dizziness research has focused on improving guideline-concordant care among clinicians, with little focus on developing patient-centered interventions to improve dizziness-related disability.

**Objective:**

To examine the feasibility of ED vestibular rehabilitation therapy (ED-VeRT) using a protocolized diagnostic classification algorithm and collection of longitudinal patient-reported outcomes.

**Design, Setting, and Participants:**

A pilot nonrandomized clinical trial of ED-VeRT vs usual care for patients presenting to the ED with dizziness at a single urban US ED was conducted from November 16, 2021, to February 6, 2023, with collection of 3-month outcomes through May 1, 2023. Patients were allocated to ED-VeRT or usual care at the discretion of the treating physician.

**Interventions:**

Use of ED-VeRT was delivered by an ED physical therapist via a protocolized diagnostic classification and treatment algorithm based on a diagnosis of benign paroxysmal positional vertigo, triggered undifferentiated dizziness, spontaneous undifferentiated dizziness, or unilateral peripheral hypofunction.

**Main Outcomes and Measures:**

Feasibility outcomes included participant screening, enrollment, and retention rates to inform the design of a future randomized clinical trial; retention was defined as completing any of 4 follow-up surveys over 3 months. The primary efficacy outcome was change in the Dizziness Handicap Inventory score; the secondary efficacy outcome was change in the Vestibular Activities Avoidance Inventory-9 score.

**Results:**

Of 366 patients screened, 125 participants were enrolled (median age, 52 [IQR, 40-66] years, 73 [58%] female, 61 [49%] White), and 105 retained (84.0%) in longitudinal data collection. Sixty-three participants (50.4%) received ED vestibular therapy and were assigned to primary diagnostic classifications of benign paroxysmal positional vertigo (23 [37.1%]), triggered undifferentiated dizziness (14 [22.6%]), spontaneous undifferentiated dizziness (14 [22.6%]), or unilateral peripheral hypofunction (9 [14.5%]). Despite having higher Dizziness Handicap Inventory and Vestibular Activities Avoidance Inventory scores at baseline, ED-VeRT participants reported lower dizziness handicap (difference: −1.68; 95% CI, −11.30 to 7.90) and vestibular activities avoidance (difference: −2.27; 95% CI, −8.40 to 3.86) at 3 months, although these differences were not statistically significant.

**Conclusions and Relevance:**

In this nonrandomized clinical trial, ED vestibular therapy was feasibly delivered to patients presenting to the ED with undifferentiated dizziness symptoms. For participants receiving vestibular therapy the findings for dizziness-related disability over 3 months were not statistically significant, pointing to the need for a fully powered randomized clinical trial.

**Trial Registration:**

ClinicalTrials.gov Identifier: NCT05122663

## Key Points

**Question:**

What is the feasibility of offering vestibular therapy for patients presenting to the emergency department (ED) with dizziness and collecting longitudinal patient-reported outcomes?

**Findings:**

In this nonrandomized clinical trial of 125 patients with dizziness, ED physical therapists successfully applied a protocolized diagnostic classification and treatment algorithm. Patients receiving ED vestibular therapy reported greater improvements in dizziness handicap, vestibular activities avoidance, and sedating medication use during 3 months of follow-up, although the differences were not statistically significant in this pilot trial.

**Meaning:**

The findings of this trial suggest that ED vestibular therapy is feasible and may improve patient-reported dizziness symptoms over time; this pilot trial establishes the need for a fully powered randomized clinical trial of ED vestibular therapy for dizziness and vertigo.

## Teaser

This nonrandomized clinical trial evaluates the use of vestibular therapy administered in the emergency department to patients presenting with dizziness.

## Introduction

Dizziness is a common problem affecting an estimated 36.8 million US individuals annually^1^ and accounting for nearly 2 million annual visits to the emergency department (ED). Dizziness presents a substantial diagnostic challenge for clinicians because of its broad differential including both pathologic (eg, cerebellar stroke) and benign (eg, benign paroxysmal positional vertigo [BPPV]) causes. Thus, dizziness-related ED visits commonly prompt clinicians to pursue low-value diagnostic imaging (eg, routine computed tomography [CT] for triggered episodic vestibular syndrome^2^) and potentially unnecessary hospitalization, which in turn contribute to increased health care costs.^3,4,5^

Previous dizziness research has focused on improving the diagnostic skills of clinicians by increasing the uptake of specific examination techniques, such as the Dix-Hallpike test or Head Impulse, Nystagmus, Test of Skew (HINTS) examination,^6,7,8,9,10,11,12^ or implementing guidelines to promote standardized care.^2^ To our knowledge, no study has yet to characterize dizziness symptoms following an ED visit and only 1 study has explored patient-centered outcomes.^13^ Given this, we have little understanding of how patients fare after an ED visit for dizziness or what ED-based interventions might improve dizziness-related functioning. Emergency clinicians therefore have little guidance to offer patients regarding expected time to symptom resolution or potential treatments.

Conversely, outpatient physical therapy for dizziness and vertigo (ie, vestibular rehabilitation) has a strong evidence base.^14,15,16,17^ However, outpatient clinics differ greatly from the emergency care setting, as patients seen in outpatient vestibular therapy settings have typically received a provisional diagnosis by a referring clinician and have experienced a longer duration of symptoms, which contributes to greater diagnostic clarity. Whether vestibular rehabilitation can be delivered effectively by physical therapists in the ED—where patients present with acute-onset dizziness symptoms and frequently have received little to no prior medical evaluation—is currently unknown.^18,19,20^

We therefore conducted a prospective nonrandomized pilot trial to obtain feasibility data from an ED vestibular rehabilitation intervention delivered by physical therapists and preliminary patient-reported outcomes in the 3 months following an ED visit for dizziness. We considered this work foundational to a future randomized clinical trial of an ED vestibular rehabilitation therapy intervention.

## Methods

### Study Setting

A prospective nonrandomized pilot trial, ED-VeRT was conducted at an urban academic hospital ED in Chicago, Illinois, with more than 93 000 annual visits. This trial was approved by the Northwestern University Institutional Review Board and launched on November 16, 2021. Patient participant enrollment concluded on February 6, 2023, with follow-up data collected through May 1, 2023. This study followed the Transparent Reporting of Evaluations With Nonrandomized Designs (TREND) reporting guideline^21^ and a prespecified statistical analysis plan (Supplement 1). Patients gave written consent to study participation and received a $10 gift card for study enrollment at the index ED visit and for each additional follow-up survey completed, up to a total of $50.

### Participants

Patients presenting to the ED with chief symptoms of dizziness or vertigo were assessed for study eligibility by research assistants who were present during normal business hours (Monday-Friday, 9 am-5 pm) and on select evenings (5 pm-9 pm) and weekends (10 am-6 pm). Eligibility criteria included an isolated symptom relating to dizziness or vertigo, age 18 years or older, and absence of any obvious non–balance related medical explanation for dizziness (eg, severe anemia, sepsis, and arrhythmia) as determined by the treating ED physician. We excluded patients with any severe neurologic deficit concerning for ischemic or hemorrhagic stroke (ie, that would necessitate activating a stroke code) and those who could not complete follow-up assessments in English by email or telephone.

### Intervention

Participants received either the ED-VeRT intervention or usual care at the discretion of the treating ED physician. Patients and physicians were therefore unblinded to treatment allocation. Usual care consisted of any testing or treatment not involving the ED physical therapist in accordance with the treating physicians’ usual and customary practice. This could include physical examination, therapeutic maneuvers, diagnostic imaging and laboratory testing, medication administration and/or prescribing, and patient education or reassurance.

**Figure 1.**
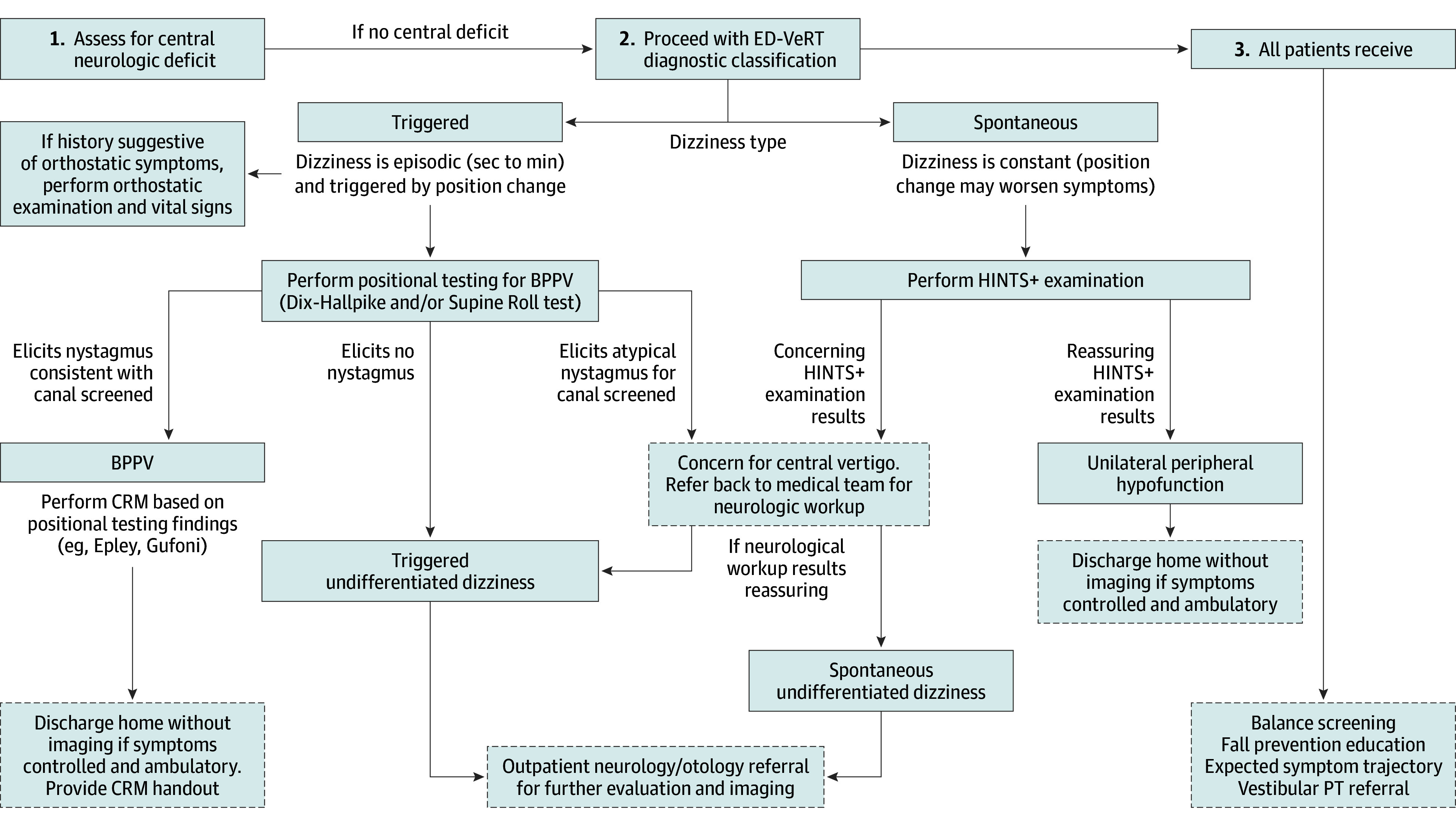
Emergency Department Vestibular Rehabilitation Therapy (ED-VeRT) Diagnostic Classification Algorithm BPPV indicates benign paroxysmal positional vertigo; CRM, canal repositioning maneuvering; HINTS+, head impulse, nystagmus, test of skew plus hearing; PT, physical therapy.

The ED-VeRT intervention consisted of a formal evaluation by an ED physical therapist who administered a protocolized diagnostic classification and treatment algorithm for dizziness and vertigo (Figure 1). The Northwestern Memorial Hospital maintains a full-time ED physical therapist for the evaluation and treatment of patients with various clinical conditions.^22,23^ The ED-VeRT algorithm classifies patients into 1 of 4 possible diagnostic categories based on key history and examination findings: BPPV, triggered undifferentiated dizziness, spontaneous undifferentiated dizziness, and unilateral peripheral hypofunction (eg, neuritis, labyrinthitis). The algorithm was developed from the Newman-Toker algorithm^24^ based on symptom timing, triggers, and targeted bedside eye examinations and initially branches based on a determination of triggered (episodic) vs spontaneous nystagmus, with subsequent positional testing for BPPV (eg, Dix-Hallpike, Roll Test) or HINTS+ examination (HINTS plus hearing) for the triggered and BPPV nystagmus types. Each diagnostic classification maps to a corresponding treatment (ie, Epley maneuver for BPPV, trial of glucocorticoids for unilateral peripheral hypofunction), and classifications that might benefit from additional vestibular rehabilitation are provided with a referral to outpatient vestibular therapy. For diagnostic classifications without specific treatment maneuvers (spontaneous undifferentiated dizziness, triggered undifferentiated dizziness), the ED physical therapist recommends referral to outpatient neurology services for further evaluation of diagnoses that require additional testing or longer symptom duration (eg, vestibular migraine). The ED-VeRT algorithm additionally accounts for atypical findings that would prompt the ED physical therapist to discuss additional testing (eg, orthostatic vitals, neuroimaging) with the treating ED physician. All patients receive balance screening, education on fall prevention and safety, guidance on expected symptom trajectory, and referral to outpatient vestibular therapy.

### Main Outcomes and Measures

#### Demographic and Clinical Characteristics

We collected demographic and clinical characteristics from participant self-report at enrollment and ED visit characteristics from the electronic medical record using a structured data collection form. Participant gender and race and ethnicity were collected by self-report. Participant gender and race and ethnicity were collected to describe sample characteristics and inform generalizability. The ED physical therapist also completed a structured case report form immediately following each encounter for participants receiving the ED-VeRT intervention.

#### Feasibility Outcomes

We collected participant screening, enrollment, and retention rates to inform sample size calculations for a future full-scale randomized trial. Retention was assessed by lost-to-follow-up, defined as completing none of 4 possible follow-up surveys. For intervention adherence, we report the proportion of case report forms completed by participating physical therapists for patients in the ED-VeRT arm and the primary diagnostic classification category (eg, BPPV) selected by the treating physical therapist within the ED-VeRT algorithm.

#### Efficacy Outcomes

We collected patient-reported outcomes at enrollment and by e-mailed REDCap^25^ surveys at 1 week, 1 month, 2 months, and 3 months (primary end point) following the index ED visit. The primary efficacy outcome measure was the Dizziness Handicap Inventory (DHI), a validated 25-item instrument quantifying patient-reported handicapping effects from dizziness among functional, emotional, and physical domains. Scores range from 0 to 100, with higher numbers indicating a greater self-perceived handicap.^26^ Secondary outcomes included the short-form Vestibular Activities Avoidance Instrument (VAAI-9), patient-reported medication use in the last 24 hours, and ED visit characteristics. The VAAI-9 is a validated 9-item instrument assessing fear avoidance beliefs with scores ranging from 0 to 54; higher scores indicate greater severity.^27^ Patient-reported medication use was assessed via an ad hoc instrument developed and piloted for a similar trial focusing on low back pain.^22,28^ Our interest was in antihistamines (meclizine, diphenhydramine, and dimenhydrinate) and benzodiazepines (lorazepam, diazepam, clonazepam, and alprazolam), which we collapsed into a composite category of sedating medications. Emergency department visit characteristics included diagnostic imaging use (CT and magnetic resonance imaging of the head or brain), visit disposition (hospitalization vs discharge), and length of stay. Additional exploratory outcomes assessed over time included the global rating of change score (range, a very great deal worse to a very great deal better [operationalized as 0-15]), a numeric rating scale assessing dizziness symptoms (range, 0-10), falls, and advanced health care use, including outpatient physical therapy, primary care physician appointments, and specialist appointments.

### Sample Size

As this was a pilot feasibility trial, the targeted sample size reflected the estimated number of participants needed to demonstrate feasibility (eg, recruitment and retention rate) and produce initial efficacy estimates (eg, mean [SD] for DHI) to inform a subsequent full-scale randomized clinical trial. We initially targeted a sample size of 100 participants, which would provide precise estimation of recruitment and retention rates such that SEs for those quantities would be less than 0.05. A sample size of 100 would further provide suitably sensitive analyses of efficacy; conservatively, this sample size could detect effects on the order of Cohen *d* = 0.4, with 80% power (α = .05). In August 2022, we increased the target enrollment to 125 to account for an interim observed 20% attrition rate.

### Statistical Analysis

We used descriptive statistics to summarize baseline participant and ED visit characteristics both overall and by treatment arm: mean (SD) or median (IQR) as appropriate for continuous variables and frequency (percentage) for categorical variables. All efficacy analyses were considered exploratory with the primary focus on obtaining initial estimates of intracluster correlation and between-group differences to inform power calculations in a future full-scale trial.

Assessments of efficacy on primary and secondary outcomes used a modified intention-to-treat (mITT) approach excluding patients who were lost to follow-up. Primary and secondary outcomes were analyzed with a generalized linear mixed model (GLMM) for repeated measures; the primary outcome and end point were prespecified as the change in DHI at 3 months. The GLMM included fixed effects for study arm (ED-VeRT vs usual care), baseline outcome score, time point, time point × arm interaction, and age and gender. The GLMM included random participant effects and random physician effects to account for both within- and between-physician variability and also allow for estimation of the physician-level intracluster correlation coefficient. However, GLMMs estimated that there was no physician-level variation in all models, which led to unstable parameter estimates, and hence physician random effects were excluded from the models. The primary contrast of interest was the comparison of model-estimated mean outcome scores at 3 months across study arms; although we emphasize the reporting of point estimates and SEs, we conducted a 2-sided Wald model type III test for the treatment effect at 3 months assuming a 5% type I error rate. As this was a pilot trial, secondary outcome analyses were deemed exploratory. All analyses were performed using R, version 4.0.4 (R Foundation for Statistical Computing).

To handle missing follow-up data, we used multiple imputation with multilevel imputation models and generating m = 40 imputations using observed data in our mITT population. Because the mITT population excludes participants lost to follow-up (ie, those who answered 0 of 4 follow-up surveys), we conducted a sensitivity analysis using data on all eligible patient participants (ie, including lost to follow-up) and combining multiple imputation with inverse probability weighting.^29^ The probability of not being lost-to-follow-up was estimated as a function of baseline characteristics and baseline measures of outcomes using a logistic regression model. Then, among our mITT imputations, GLMMs were estimated using weights inversely proportional to that probability. This method adjusts analyses for differences between the lost-to-follow-up and mITT populations and provides a measures of how sensitive mITT analyses are to excluding lost-to-follow-up patient participants. Finally, we performed 2 exploratory subgroup analyses, the first dichotomizing participant age at 65 years and the second dichotomizing symptom duration at 3 days. The older age subgroup analysis was prespecified and the acute symptom duration subgroup analysis was post hoc based on peer reviewer request.

## Results

### Demographic and Clinical Characteristics

**Figure 2.**
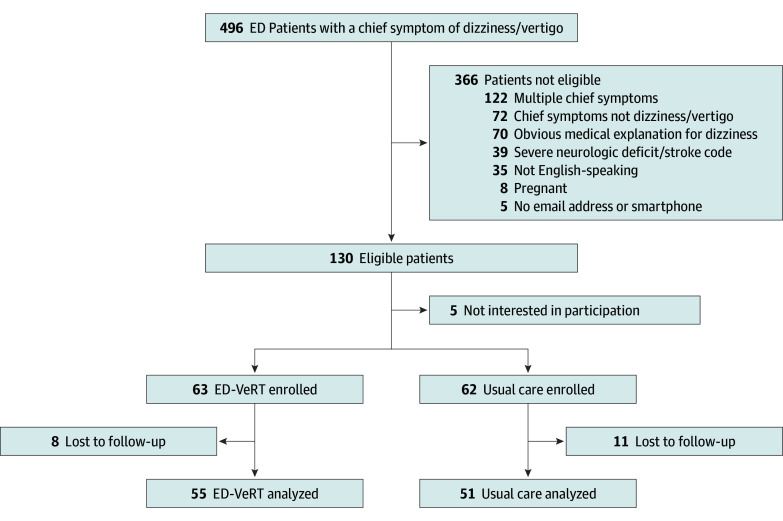
Study Flow Diagram Note that the sum of all exclusion criteria exceeds n=366 because some patients met multiple exclusion criteria. ED indicates emergency department; ED-VeRT, ED vestibular rehabilitation therapy.

We screened 366 patients for study eligibility and enrolled 125 participants (63 ED-VeRT, 62 usual care) (Figure 2). Median age was 52 (IQR, 40-66) years, 73 (58%) of the participants were female, 61 (49%) self-reported as White race, 25% as Black race, and 18% as Hispanic ethnicity (Table). Most participants reported that their dizziness symptoms had been present for less than 1 day (42%) or between 1 and 3 days (26%). Baseline outcome scores at enrollment indicated moderate disability with a median DHI score of 44 (IQR, 22-62) and a median VAAI-9 score of 34 (IQR, 23-45); participants in the ED-VeRT arm reported higher median baseline DHI and VAAI-9 scores compared with usual care participants (DHI: ED-VeRT, 48 vs usual care, 38; VAAI-9: ED-VeRT, 35 vs usual care, 30).

**Table.** Baseline Demographic, Clinical, and ED Visit Characteristics

Characteristic	No. (%)		
	ED-VeRT (n = 63)	Usual care (n = 62)	Total (n = 125)
Age, median (IQR), y	52 (41-67)	52 (38-62)	52 (40-66)
Gender			
Female	40 (63)	33 (53)	73 (58)
Male	23 (37)	29 (47)	52 (42)
Race^a^			
Asian (East Asian, South Asian)	7 (11)	6 (10)	13 (10)
Black	12 (19)	19 (31)	31 (25)
White	33 (52)	28 (45)	61 (49)
Other	3 (5)	4 (5)	7 (6)
Some other race not listed	8 (13)	5 (8)	13 (10)
Hispanic ethnicity	10 (16)	12 (19)	22 (18)
Marital status			
Married/living with partner	30 (59)	33 (62)	63 (61)
Single/unmarried	21 (41)	20 (38)	41 (39)
Highest educational level			
High school	7 (11)	8 (13)	15 (12)
College	31 (49)	32 (52)	63 (51)
Graduate or professional school	25 (40)	21 (34)	46 (37)
Insurance			
Medicaid or Medicare	27 (44)	19 (31)	46 (37)
Commercial	34 (55)	43 (69)	77 (62)
Symptom duration, d			
<1	24 (38)	29 (47)	53 (42)
1-3	16 (25)	16 (26)	32 (26)
3-7	14 (22)	10 (16)	24 (19)
≥7	9 (14)	7 (11)	16 (13)
Arrival mode			
Walked in	52 (83)	54 (87)	106 (85)
Ambulance	11 (17)	8 (13)	19 (15)
Fall due to dizziness	5 (8)	7 (11)	12 (10)
Dizziness Handicap Inventory score, median (IQR)^b^	48 (28-69)	38 (18-58)	44 (22-62)
Vestibular Activities Avoidance-9 score, median (IQR)^c^	35 (28-46)	30 (16-44)	34 (23-45)
Visit disposition			
Discharge	47 (75)	41 (66)	88 (70)
Hospitalization	3 (5)	8 (13)	11 (9)
Observation	13 (21)	13 (21)	26 (21)
ED length of stay, median (IQR), h	5.1 (3.6-8.1)	4.4 (3.1-7.4)	4.7 (3.2-7.9)
Any CT imaging	35 (56)	44 (71)	79 (63)
Any MRI	17 (27)	18 (29)	35 (28)
Any specialty consult	13 (21)	12 (19)	25 (20)

Abbreviations: ED, emergency department; ED-VeRT, ED vestibular rehabilitation therapy; CT, computed tomography; MRI, magnetic resonance imaging.

^a^ Asian race includes East Asia (eg, China, Japan, Korea) or South Asian (eg, India, Pakistan). Other race includes Hawaiian or Pacific Islander, multiple races, Native American or Alaska Native, and prefer not to disclose.

^b^ A 25-item instrument quantifying patient-reported handicapping effects from dizziness among functional, emotional, and physical domains. Scores range from 0 to 100; higher numbers indicate a greater self-perceived handicap.

^c^ A 9-item instrument assessing fear avoidance beliefs. Scores range from 0 to 54; higher scores indicate greater severity.

### ED Visit Characteristics

A total of 46 unique physicians cared for the 125 patient participants (median, 4 [IQR 2-5] patients per physician). Most patients received diagnostic imaging of the brain, with 63% receiving a CT scan and 28% receiving magnetic resonance imaging (Table); fewer participants in the ED-VeRT arm received diagnostic imaging (CT brain: 56% ED-VeRT vs 71% usual care). Most participants (70%) were discharged home from the ED; median length of ED stay for the total cohort was 4.7 (IQR, 3.2-7.9) hours. More participants in the ED-VeRT group were discharged home compared with usual care (75% vs 66%), while ED length of stay was slightly longer for ED-VeRT participants (5.1 vs 4.4 hours).

### ED-VeRT Intervention Characteristics

The 63 participants in the ED-VeRT group were evaluated by 5 unique ED physical therapists using the ED-VeRT diagnostic classification algorithm; 1 physical therapist performed most of these evaluations 49 [77.8%]). All ED-VeRT participants had a corresponding case report form completed by the treating ED physical therapist. The most common ED-VeRT classification assigned was BPPV (23 [37.1%]), followed by triggered undifferentiated dizziness (14 [22.6%]), spontaneous undifferentiated dizziness (14 [22.6%]), and unilateral peripheral hypofunction (9 [14.5%]). Two participants were classified as other due to their inability to fully complete the ED-VeRT evaluation. The first patient declined participation in most bedside testing maneuvers due to symptom severity; the physical therapist’s best classification was spontaneous undifferentiated dizziness. The second participant reported complete symptom resolution by the time of the physical therapist evaluation. Of the 23 participants classified as having BPPV, 21 were classified as unilateral posterior canal and 2 as unilateral horizontal canal. All 23 BPPV participants received a canalith repositioning maneuver; 1 of the participants with unilateral posterior canal converted to unilateral horizontal canal with a canalith repositioning maneuver. The 62 participants in the usual care group were not evaluated by the ED physical therapist and therefore did not receive an ED-VeRT diagnostic classification.

###  Longitudinal Outcomes

**Figure 3.**
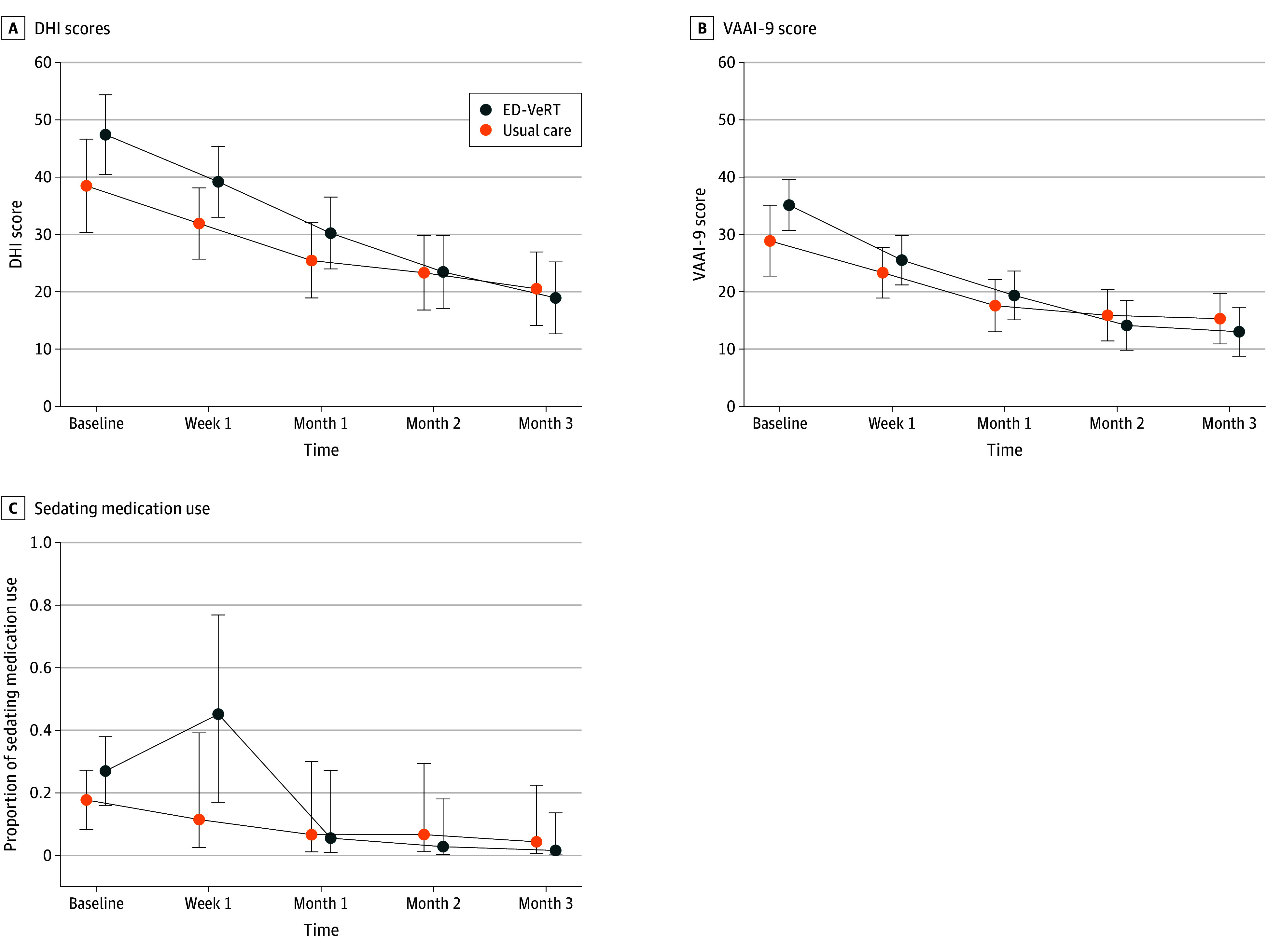
Longitudinal Patient-Reported Outcomes Mean Dizziness Handicap Inventory (DHI) scores (A), Vestibular Activities Avoidance Inventory-9 (VAAI-9) scores (B), and sedating medication use (C) over time. Error bars indicate 95% CIs calculated under the normal distribution assumption. Note that baseline values are calculated directly from observed data, whereas follow-up values are estimated by the generalized linear mixed model. ED-VeRT indicates emergency department vestibular rehabilitation therapy.

A total of 105 participants (84%) provided follow-up data for at least one time point and were retained in the longitudinal model. Figure 3 shows adjusted model-estimated means for DHI, VAAI-9, and sedating medication use at each study time point; eFigure 1 and eFigure 2 in Supplement 2 show adjusted model-estimated means for numeric rating scale and global rating of change scores.

At the primary end point of 3 months, for GLMM estimated ED-VeRT participants compared with usual care participants, the DHI group difference for dizziness handicap was −1.68 (95% CI, −11.30 to 7.90) and the VAAI-9 group difference for vestibular activities avoidance was −2.27 (95% CI, −8.40 to 3.86); sedating medication use for ED-VeRT was 6% compared with 11% for usual care (odds ratio [OR], 0.49; 95% CI, 0.10-2.48). Use of sedating medications at the 1-week time point for ED-VeRT participants was 34% vs 17% for usual care (OR, 2.45; 95% CI, 0.68-8.77). The ED-VeRT participants had similar 3-month numeric rating scale (difference: −0.03; 95% CI, −0.96 to 0.91) and slightly higher global rating of change scores (difference: 0.18; 95% CI, −1.00 to 1.30) compared with usual care participants, although the differences were not significant. eTable 1 in Supplement 2 presents group means for all longitudinal outcomes at each follow-up time point.

Reductions for ED-VeRT participants compared with usual care participants was −6.63 (95% CI, −17.62 to 4.37; *P* = .24) for DHI scores and −4.30 (95% CI, −11.50 to 2.90; *P* = .24) for VAAI-9 scores; the relative odds ratio for sedating medication use was 0.18 (95% CI, 0.03 to 1.30; *P* = .10). For the exploratory outcomes over 3 months, reductions for ED-VeRT participants compared with usual care participants was −0.76 (95% CI, −1.90 to 0.39; *P* = .19) for the numeric rating scale and 0.17 (95% CI, −1.30 to 1.64; *P* = .82) for global rating of change. The overall time × group interaction term was not statistically significant for DHI score (*P* = .67), VAAI-9 score (*P* = .72), or sedating medication use (*P* = .31). Physician-level intracluster correlation coefficients for DHI and VAAI-9 had *P* < .001, indicating almost no between-physician variation; because this led to unstable parameter estimation, the physician random effect was omitted from the final models. Full model summaries are presented in eTable 2 in Supplement 2.

### Sensitivity and Subgroup Analyses

Sensitivity analyses yielded qualitatively similar point estimates and 95% CIs (eTable 3 in Supplement 2). In analyses combining multiple imputation and inverse probability weighting, the difference between the ED-VeRT and usual care groups at the 3-month primary end point was −1.86 (95% CI, −11.50 to 7.74) for DHI and −2.39 (95% CI, −8.50 to 3.72) for VAAI-9. In evaluation of outcomes over 3 months of follow-up, reductions for ED-VeRT participants compared with usual care participants was −7.02 (95% CI, −18.00 to 3.98) for DHI and −4.46 (95% CI, −11.50 to 2.56) for VAAI-9.

Subgroup analyses for older age and acute symptom duration are reported in eTable 4 and eTable 5 in Supplement 2. The ED-VeRT effect sizes for DHI and VAAI-9 were more pronounced among the subgroups of age less than 65 years and symptom duration of 3 days or less but was not statistically significant.

### Health Care Use Outcomes of Interest

During 3 months of follow-up, 3 participants (5.7%) in the ED-VeRT group and 2 (3.9%) in the usual care group reported a fall. Eight participants (15.0%) in the ED-VeRT group and 3 usual care participants (5.9%) reported a repeat ED visit; 3 participants in each of the ED-VeRT (5.8%) and usual care (5.9%) groups reported a subsequent hospitalization. Twenty-six ED-VeRT participants (49.1%) and 11 usual care participants (21.6%) reported following up with an outpatient physical therapist. Rates of outpatient follow-up with primary care professionals (21 [40.4%] ED-VeRT, 21 [41.2%] usual care) and specialists (16 [30.8%] ED-VeRT, 16 [31.4%] usual care) were similar.

## Discussion

In this pilot nonrandomized trial of an ED vestibular therapy intervention, we enrolled 125 patients presenting to the ED with dizziness or vertigo and characterized patient-reported dizziness symptoms over 3 months. Participants evaluated by the ED physical therapist were classified using the ED-VeRT diagnostic algorithm and had similar ED lengths of stay compared with participants receiving usual care, with more frequent discharge to home and lower use of diagnostic imaging. Although participants receiving ED-VeRT had higher baseline symptom scores, likely reflecting the nonrandomized study design, differences in dizziness handicap scores at 3 months were not statistically significant compared with participants receiving usual care.

Because the purpose of this pilot trial was to justify and inform the design of a subsequent randomized clinical trial, we emphasize the study’s feasibility findings relating to intervention delivery and participant enrollment and retention. We found that patients presenting in the ED with dizziness or vertigo could be successfully classified by physical therapists using the ED-VeRT diagnostic classification algorithm, and that approximately half of the participants were placed in the diagnostic classifications of BPPV (37.1%) or unilateral peripheral hypofunction (14.5%), while the other half were classified as undifferentiated dizziness of either a spontaneous or triggered nature. Additionally, we demonstrate that patients presenting to the ED with undifferentiated dizziness can be enrolled and retained in longitudinal follow-up. The data generated from this pilot study may subsequently inform the design and sample size calculations of a multisite randomized clinical trial of ED vestibular therapy for dizziness and vertigo.

The patient experience of dizziness represents a heterogeneous list of differential diagnoses, including conditions that are potentially amenable to an intervention delivered in an emergency care environment (eg, Epley maneuver for BPPV, corticosteroids for vestibular neuritis [unilateral peripheral hypofunction])^30,31^ to nonspecific or chronic conditions (eg, persistent postural perceptual dizziness) that are difficult to diagnose and treat in a limited acute care encounter. To complicate matters further, the term dizziness is frequently used by patients to describe various general states of feeling unwell. Although we attempted to exclude patients with obvious nonvertiginous causes of dizziness, such as fever or severe anemia, and included consideration of orthostatic symptoms in the ED-VeRT algorithm, the general tendency to describe various states of unwell as dizziness likely contributes to the classification of some patients into undifferentiated triggered or spontaneous dizziness categories. Patients with undifferentiated dizziness classifications may still benefit from the general education, safety teaching, and diagnostic reassurance provided by a physical therapist, as a previous study observed how patients evaluated by an ED physical therapist for low back pain described benefits from the increased time and attention provided by an expert.^32^

Ideally, we would compare the effect of ED vestibular therapy vs usual care among specific subgroups of diagnostic classifications (eg, BPPV, unilateral peripheral hypofunction), but because usual care participants did not receive the ED-VeRT standardized diagnostic classification algorithm, we were not able to assemble comparator subgroups. Although one might suggest that physician assessments (as documented in final billing diagnoses or physician notes) could be used to specify dizziness subgroups, ED visit diagnoses tend to be symptom-based (eg, *International Statistical Classification of Diseases and Related Health Problems, 10th Revision* [*ICD-10*] code R42: dizziness) rather than specific diagnoses (eg, *ICD-10* code H81.09 Meniere disease), and physician assessments of dizziness in clinical notes have poor reliability and accuracy.^33^ This latter point about physician inconsistency in evaluating the patient symptom of dizziness has led to a preponderance of clinician-oriented educational interventions in the extant dizziness literature, which tends to emphasize simplified assessment tools (eg, HINTS) or clinical decision supports in pursuit of a clinician-oriented outcome, such as a decreased miss rate for stroke. Clinician-oriented interventions tend to target overuse of low-value care in the diagnostic workup for dizziness symptoms (routine CT for triggered episodic vestibular syndrome^2^), which we also observed in this study: 63% of study participants received a CT scan, 28% received magnetic resonance imaging.

This present study represents a notable departure from the extant dizziness literature as it focuses on the patient-oriented outcome of dizziness-related disability. Most previous dizziness research has focused on the clinician-centric goal of excluding the rare diagnosis of posterior circulation stroke, with relatively little attention to improving the patient’s understanding of their dizziness symptoms or reducing their symptom burden following ED discharge.^6,7,8,9,10,11,12^ While we acknowledge that minimizing the miss rate of catastrophic neurologic diagnoses is a paramount goal, this current work also asks the critically important patient-centered question: how do we actually improve the care trajectory and symptom burden of patients who present to the ED with severe dizziness and vertigo symptoms? To that end, this study is an important first step in suggesting the substantial value of ED vestibular therapists in patient recovery by reducing dizziness handicap and vestibular activities avoidance.

### Limitations

This study is limited by its nonrandomized design, as patient allocation to ED-VeRT vs usual care was based on physician discretion and ED-VeRT participants reported higher symptom scores at baseline compared with those receiving usual care. Although we adjusted for baseline outcome scores in our model, other unmeasured variables may have contributed to additional confounding that was not accounted for in our statistical model, and our primary analytic approach excluded participants who provided no follow-up data. Moreover, as efficacy was not of a primary interest in this study, the sample size was small and analyses were likely underpowered. Finally, a single physical therapist performed most vestibular evaluations, which may limit generalizability of these findings.

## Conclusions

In this pilot nonrandomized clinical trial of ED vestibular rehabilitation for dizziness and vertigo, we observed the feasibility of an ED physical therapy intervention using a standardized diagnostic classification system and generate initial point estimates of longitudinal patient-reported outcomes during 3 months of follow-up. The findings of this nonrandomized clinical trial point to a need to conduct a fully powered randomized clinical trial of ED vestibular therapy.

## Notes

Supplement 1.
Trial Protocol

Supplement 2. **eFigure 1.** Numeric Rating Score (NRS) Over Time **eFigure 2.** Global Rating of Change (GROC) Score Over Time **eTable 1.** Model-Adjusted Means at Each Follow-Up Timepoint **eTable 2.** Full Model Summary **eTable 3.** Sensitivity Analysis Using Multiple Imputation and Inverse Probability Weighting **eTable 4.** Subgroup Analysis for Age: Model-Adjusted Means at Each Follow-Up Timepoint **eTable 5.** Subgroup Analysis for Symptom Duration: Model-Adjusted Means at Each Follow-Up Timepoint

Supplement 3.
Data Sharing Statement
