# Supplementary material for: Emergency Department Vestibular Rehabilitation Therapy for Dizziness and Vertigo: A Nonrandomized Clinical Trial
Source: JAMA Netw Open. 2025 Feb 14;8(2):e2459567. doi: 10.1001/jamanetworkopen.2024.59567 (PMC11829232; doi:10.1001/jamanetworkopen.2024.59567)
Supplement: Supplement 1. — Trial Protocol [file jamanetwopen-e2459567-s001.pdf]

STATISTICAL ANALYSIS PLAN (SAP)

A Pilot Feasibility Trial of Emergency Department Vestibular Rehabilitation Therapy for Dizziness and Vertigo (ED-VeRT)

October 19, 2022

Version 1.0

NU IRB: STU00215025
Registration: NCT05122663

STATISTICAL ANALYSIS PLAN (SAP)

A Pilot Feasibility Trial of Emergency Department Vestibular Rehabilitation Therapy for Dizziness and Vertigo (ED-VeRT)

Principal Investigator: Howard S. Kim, MD MS
Co-Investigators: Heidi R. Roth, PT DHS; Peter B. Pruitt, MD MS; Danielle M. McCarthy, MD MS; Jody D. Ciolino, PhD; Jacob M. Schauer, PhD

1. INTRODUCTION

This document outlines the rationale for ED-VeRT and planned analyses. Briefly, ED-VeRT is an observational trial evaluating the feasibility of an embedded emergency department (ED) physical therapy intervention for dizziness and vertigo. Individual ED patients will be consented and enrolled during their index ED visit and will be followed to a primary endpoint of three months. We will compare a number of outcomes among participants who were evaluated by an ED physical therapist versus usual care (i.e., not evaluated by an ED physical therapist), as determined by the treating ED physician in the course of their typical clinical practice. Participants will provide follow-up data at one week, one month, two months, and three months following the index ED visit.

2. STUDY OUTCOMES

Primary Efficacy Outcome:
The primary efficacy outcome is the change in **Dizziness Handicap Inventory (DHI) score** at three months after the index ED visit. The DHI is a 25-item patient-reported outcome measure quantifying the impact of dizziness on daily life and self-perceived handicap, with distinct physical, emotional, and functional subdomains. Scores range from 0-100, with higher scores indicating greater disability.

Secondary Outcomes
Secondary efficacy outcomes include:

1) **Vestibular Activities Avoidance Instrument (VAAI-9)** at three months (change in score from baseline to three months). The VAAI-9 identifies fear avoidance beliefs in person with dizziness. The VAAI-9 is the 9-item short form version of the VAAI.
2) **Patient-Reported Medication Use in the last 24 hours.** This will be collected using a customized instrument assessing whether participants have taken any medications for their dizziness symptoms in the last 24 hours. The 24-hour timeframe was selected to maximize accuracy in patient recall and has been used previously. In brief, medications are listed by brand and generic names; a "yes" response to any medication triggers an additional query asking the participant to specify the medication dose (e.g., diazepam 5mg) and quantity (e.g., four pills), allowing for standardization. We anticipate treating this variable as either count or a binary (any dose vs. none), or continuous (MME) for analyses. We plan to focus on anti-histamines and benzodiazepines as medications of interest.
3) **ED Diagnostic Imaging Utilization** at the index ED visit. This will include the proportion of ED visits in which any diagnostic imaging of the brain was performed, including computed tomography and magnetic resonance imaging.
4) **ED Length of Stay** at the index ED visit. Total length of stay is calculated from the time the patient is placed in a room to the time a patient is discharged from the room. We will analyze this outcome separately for ED visits resulting in discharge vs hospitalization (including inpatient and observation).

Exploratory Outcomes
We expect the following outcomes to be related to the primary and the major secondary outcomes of interest. We deem the more exploratory in nature, and they thus carry less weight in analyses and overall inferences regarding efficacy of intervention.

1) **Benzodiazepine Prescription Filling** will be queried in the state prescription monitoring database. We anticipate treating this variable as count, binary, or continuous (MME).
2) **Numeric Rating Scale (NRS)** measures symptom intensity from 0 to 10 and is easily understood by laypersons, clinicians, and researchers. We will assess a single item relating to average dizziness intensity over the last 24 hours. We plan to treat this as a continuous outcome, but we anticipate

requiring transformation or nonparametric analyses, as this variable will likely be skewed and exhibit flooring / ceiling effects.

3) **Global Rating of Change (GROC)** is a single-item survey widely used by clinicians and researchers to quantify functional disability and evaluate the overall effectiveness of therapy. This item ranges from zero (a very great deal worse) to 14 (a very great deal better). We plan to initially treat this measure as continuous, but we anticipate exploring this outcome as a count variable, requiring transformation, or using nonparametric analyses.
4) **Advanced Healthcare Resource Utilization.** We will assess the proportion of participants who utilized advanced healthcare resources for dizziness after their index ED visit, defined as advanced imaging (e.g., magnetic resonance imaging) or procedures/surgery (e.g., epidural steroid injection, lumbar discectomy).
5) **Follow-up with outpatient physical therapy.** We will assess the proportion of participants who report outpatient physical therapy follow-up for vestibular rehabilitation.
6) **Intervention fidelity:** We will assess whether participants in the ED PT arm can be classified into the ED-VeRT intervention algorithm. This algorithm uses components of the physical exam to guide the physical therapist into classifying the patient into one of five classifications: orthostatic, benign paroxysmal positional vertigo (BPPV), triggered undifferentiated dizziness, spontaneous undifferentiated dizziness, and unilateral peripheral hypofunction. The treating ED physical therapist will complete a PT Assessment form on the day of the index ED visit indicating the patient's ED-VeRT classification (Figure 1).

Figure 1: ED-VeRT Intervention Algorithm

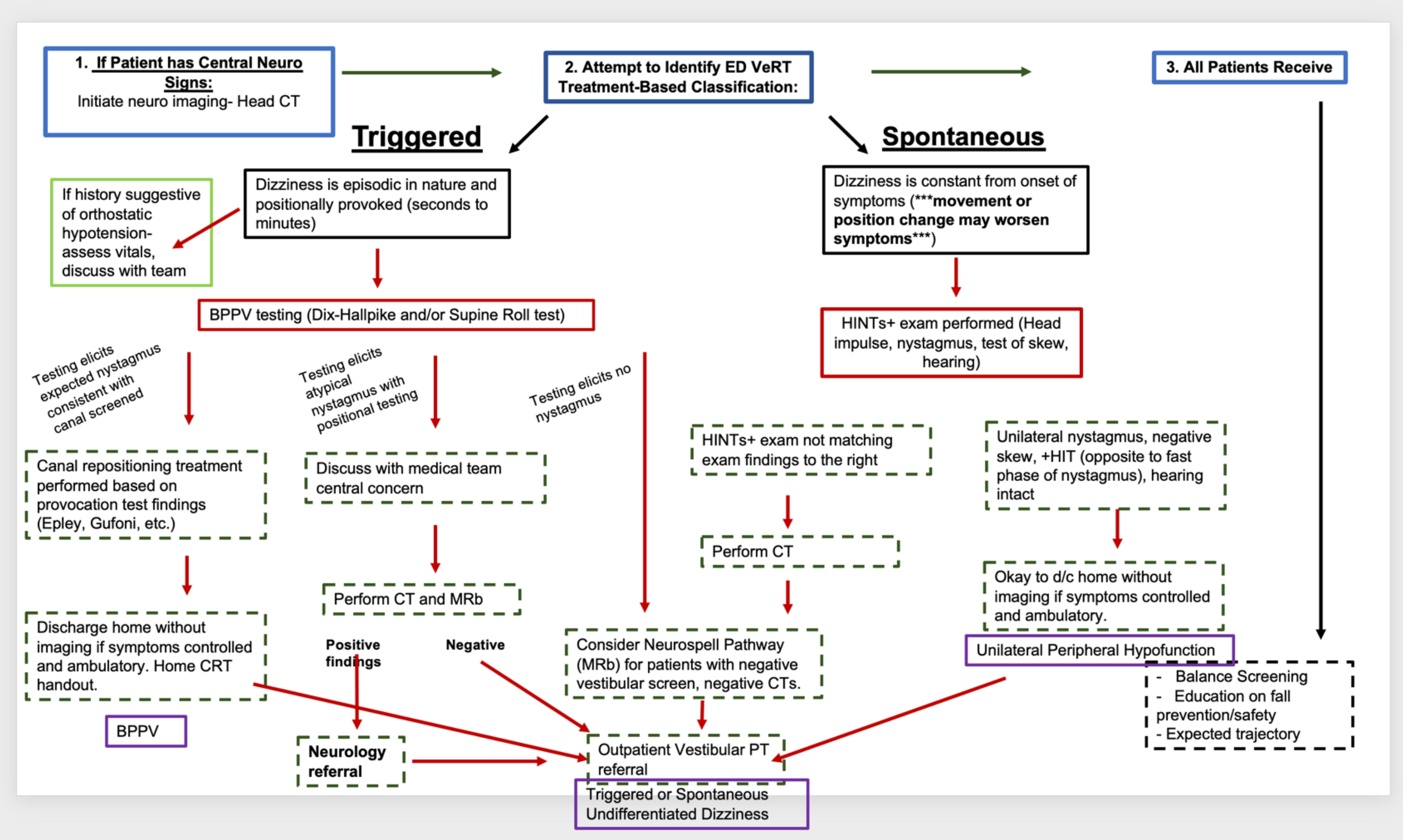

3. DEMOGRAPHICS AND BASELINE ASSESSMENTS

We will collect and summarize the following baseline demographic and clinical characteristics:

1) Sex and gender
2) Race and ethnicity
3) Education level
4) Marital status
5) Employment status
6) Income level
7) Symptom duration
8) Fall as a result of dizziness
9) Previous healthcare encounters for this episode of dizziness
10) Primary diagnosis
11) Medications administered / prescribed during initial ED visit
12) Insurance type
13) Documentation of common physical exam assessments (nystagmus, Dix-Hallpike, HINTS components) and interview questions (room-spinning sensation) in the clinical note.

Note that some additional exploratory analyses may examine these additional variables as covariates and/or effect modifiers as well. We will label any exploratory analyses involving additional potential covariates as post hoc in any dissemination materials.

4. DATA STORAGE

Data will be collected and managed using Research Electronic Data Capture (REDCap) housed at Northwestern University's Clinical and Translational Sciences Institute (CTSA), NUCATS. REDCap is a secure, web-based application designed for research studies that provides an intuitive interface for validated data entry, audit trails for tracking data manipulation and export procedures, and automated export procedures for seamless data downloads to common statistical packages, and procedures for importing data from external sources. Individualized REDCap survey links will be sent to participants using REDCap-initiated emails.

5. RANDOMIZATION METHODS

This is a non-randomized study. Patients will be assigned to the ED physical therapy or usual care arm based on the clinical course of care as determined by the treating ED physician. Participants are enrolled into this study following this treatment decision.

6. STATISTICAL METHODS

We plan to use descriptive statistics to summarize baseline patient and physician-level variables both overall and by arm. We will use mean±standard deviation (or median and interquartile range [IQR] as appropriate) for continuous variables and frequency / percentage for categorical variables. Specifically, we will summarize age, sex, baseline patient-reported outcome scores (DHI and VAAI-9), and the variables listed above. Analyses will involve normal theory methods in general, and in cases of violations of assumptions, we will consider transformation and / or nonparametric / exact methods as appropriate.

Analyses will assume a two-sided 5% significance level. We do not plan to control for multiple hypothesis tests but will explicitly label analysis of non-primary outcomes as exploratory in nature. In analyses for each outcome, we plan to control for the respective outcome value at baseline (i.e., in an analysis of covariance [ANCOVA] approach). Analyses for the primary outcome (Y) will involve a linear mixed model (LMM) with repeated measures with fixed effects for: study arm, baseline outcome score (Y0), timepoint, timepoint-by-arm interaction, and known influential predictor effects (age, sex). Inference will focus on treatment impacts for the outcome at three months. We will include a random physician effect to account for both within and between physician variability and also to allow for estimation of the intra-cluster correlation coefficient (ICC). The repeated measures on the same participant over time will also introduce a correlation structure across time points, providing the justification for modeling the correlation structure at the participant level over time. We will

use an unstructured correlation matrix to account for the repeated measures within a participant as this has the least assumptions. If the model does not converge or parameters cannot be estimated under this unstructured covariance pattern, we will explore simpler covariance patterns using residual estimated maximum likelihood (REML) comparisons. Including repeated measures per participant will allow us to make most use of all participant data after baseline. We will use assume an unstructured covariance across time.

To evaluate efficacy, the Wald model type III test for fixed arm effect will be evaluated assuming a two-sided 5% type I error rate. The primary contrast of interest to address the primary research aims involves the comparison of the model-estimated mean outcome score at three months (T4) across study arms. This modeling strategy is robust to unbalanced (i.e., incomplete) data across study time points. We will also provide results for unadjusted analyses (i.e., without accounting for the pre-specified covariates). Analyses of additional outcomes will follow the same general analytic strategy: LMM with fixed arm, baseline outcome value, influential baseline covariate effects, and a random physician effect and covariance patterns to account for repeated measures within participants. We chose to incorporate baseline outcome as a covariate in the model, rather than as a time point, based on clinical reasoning. As these baseline values (e.g., DHI score at the index ED visit) are assessed pre-intervention and primary analyses aim to assess outcome(s) as follow-up accounting for pre-intervention state. Incorporating this baseline value in the analytic model as a fixed effect will increase precision and reduce bias on the intervention effect estimate for primary outcome at the time point of interest as the baseline value will likely be highly correlated with outcome at follow-up.

Residual diagnostics will assess model fit and assumptions, and in the case of violation, we will explore transformations / nonparametric methods as indicated above. In the event of poor model fit, we may explore different distributional assumptions as appropriate (e.g., Poisson for count or rate data) with the corresponding canonical link (e.g., log) function. As above, we will assess model fit via residual diagnostics and may consider transforming or nonparametric methods as needed.

Analyses for outcomes that are either binary or count will follow the same general approach as above; however, they will involve generalized linear mixed effects (GLMMs) models with the appropriate distributional (e.g., binomial or Poisson) and link (e.g., logit or log) assumptions. Modeling the covariance structure for these outcomes may result in unstable model estimates. If this occurs, we anticipate removing the random physician effect and including a random participant effect instead to account for correlation.

<u>Exploratory Analyses</u>
In addition to repeating the above analyses with exploratory outcomes, we will conduct exploratory analyses to study effects among subgroups of patients (moderator analyses) and examine the potential impact of PT use among patients in the control arm. Planned moderator analyses will include the following moderators:

1. Initial symptom burden of “moderate/severe,” as determined by DHI score ≥36
2. Age ≥ 65 years old
3. Primary ED-VeRT algorithm classification (orthostatic, BPPV, triggered undifferentiated dizziness, spontaneous undifferentiated dizziness, unilateral peripheral hypofunction)

Analyses will focus on DHI scores measured three months after the index ED visit, as well as the secondary and exploratory outcomes at the same time point. Analyses will involve generalized linear models with appropriate link functions (identity for DHI and logit for medication use) that include fixed effects for baseline outcomes measures, treatment assignment, a moderator variable, and a treatment-moderator interaction. As above, DHI will be modeled with standard normality assumptions, which will be evaluated via residual diagnostics and appropriate transformations will be used as necessary. Separate models will be fit for each outcome and moderator. Tests for the treatment-moderator interaction will be two-sided with a 5% type I error rate, and we will report point estimates and 95% confidence intervals. For the logistic regression involving medication use, we will use Wald confidence intervals and Wald tests.

Mediation analyses will focus on VAAI-9 as a possible mediator. Our hypotheses are that ED PT evaluation is associated with reductions in vestibular avoidance, which will in turn lead to lower reported dizziness handicapping and medication use. Our key dependent variables will be DHI and medication use at three months after the index visit. VAAI-9 measured at one month will be the mediator of interest. We will use a nonparametric approach to analyses, running separate models for each outcome and mediator (1). In addition,

we will examine the possible correlation between mechanisms by using a joint nonparametric estimation framework (2).

Feasibility Analyses

As the goal of this pilot trial is to justify and inform the design of a future randomized clinical trial, we anticipate conducting several feasibility assessments. Analysis will be primarily descriptive for use in future trial planning.

1) **Intervention Fidelity.** We will describe the total number of participants in the ED PT arm that are able to be classified into each respective ED-VeRT algorithm classification**:** orthostatic, benign paroxysmal positional vertigo (BPPV), triggered undifferentiated dizziness, spontaneous undifferentiated dizziness, and unilateral peripheral hypofunction. We will focus on classifications into the BPPV and unilateral peripheral hypofunction (e.g., vestibular neuritis) categories given that they represent specific diagnoses that can be matched to specific vestibular interventions. These data will be used to describe the appropriateness, feasibility, and fidelity of the intervention for use in a future randomized trial.
2) **Participant recruitment and retention rate**. Recruitment rate is defined as the number of participants enrolled per week, and the proportion of participants who are enrolled in the study among potential participants screened for enrollment. Retention rate is defined as the proportion of participants who provide follow-up data at the primary study endpoint (three months), and the proportion of participants who provide any follow-up data at any of the follow-up timepoints (one week, one month, two months, and three months after the index ED visit). These data will be used in sample size and power calculations of a future trial, as well as logistical planning (e.g., trial duration, number of sites).
3) **Intra-cluster correlation.** We will additionally explore whether there is evidence of a nonzero intra-cluster correlation (ICC; i.e., a physician effect) for participant-level outcomes; this will again increase precision for future power and sample size calculations for the subsequent randomized trial. Though given the nature of the intervention and outcomes, we envision the participant-level covariates will explain more variation in outcomes than any physician-level effects. Therefore, we will attempt to estimate ICC for outcomes, but we do not anticipate the ICC to be estimable after accounting for baseline covariates.

7. ANALYTIC DATASET

Primary and secondary outcomes will be evaluated across arms under a modified intention-to-treat (mITT) principle, whereby only participants contributing at least one follow-up data point will be included. Sensitivity analyses will be detailed after data collection; however, we plan to conduct sensitivity analyses that would involve:

1) Excluding patients who are ultimately admitted to the hospital after their ED visit (including both inpatient and observation stays)
2) Excluding patients with an alternative diagnosis after enrollment that would have deemed them otherwise ineligible
3) Excluding ED PT arm participants in whom the physical therapist evaluation was ordered as part of inpatient workup (i.e., timestamp for PT order occurred after the bed request order)

In the event of large amounts of missing data (i.e., more than 10%), multiple imputation analyses will be explored. We will examine rates of missing data for all variables and determine whether the rates vary by participant characteristics, etc. These summarizations will inform potential biases resulting from missing data. Mixed effects models planned for longitudinal analysis are generally robust for unbalanced data across study time points. Additional sensitivity analyses may be explored to evaluate overall trial robustness. These analyses will again serve as sensitivity analyses to the primary analyses, and the details of these analyses will be documented at the time of analyses (if needed).

8. POWER AND SAMPLE SIZE CONSIDERATIONS

As this is a pilot feasibility trial, the planned sample size (n=125) reflects the total number of participants needed to produce initial estimates of efficacy (e.g., mean/standard deviation for DHI) and feasibility (e.g., recruitment/retention rate) for the planning of a subsequent randomized clinical trial. Based on previous work

utilizing similar patient-reported outcome instruments in the ED, we expect a 20% attrition rate, thus leaving a total of n=100 participants in the modified intention-to-treat analysis. Importantly, the enrollment target of n=125 does *not* reflect a sample size that has been adequately powered to detect meaningful differences in the clinically important outcomes.

9. TECHNICAL DETAILS

The SAP is subject to version control, and we anticipate modifications to analytic plans be documented herein. As in any study, the analytic plan may change due to assumption violations, logistical issues, unexpected empirical distributions of study outcomes, or a combination thereof. In these cases, the SAP will be updated accordingly. All analyses will be performed via SAS version 9.4 or higher (The SAS Institute; Cary, NC) or R version 4.0.4 or higher (The R Foundation for Statistical Computing platform). Table and figure formatting and style may be dictated by mode of dissemination or specific target journal(s) for results dissemination.

References:

1. Imai K, Keele L, Yamamoto T. Identification, inference and sensitivity analysis for causal mediation effects. Statistical science. 2010;25(1):51-71.
2. Imai K, Yamamoto T. Identification and sensitivity analysis for multiple causal mechanisms: Revisiting evidence from framing experiments. Political Analysis. 2013;21(2):141-71.
