# Supplementary material for: Emergency Department Vestibular Rehabilitation Therapy for Dizziness and Vertigo: A Nonrandomized Clinical Trial
Source: JAMA Netw Open. 2025 Feb 14;8(2):e2459567. doi: 10.1001/jamanetworkopen.2024.59567 (PMC11829232; doi:10.1001/jamanetworkopen.2024.59567)
Supplement: Supplement 2. — eFigure 1. Numeric Rating Score (NRS) Over Time eFigure 2. Global Rating of Change (GROC) Score Over Time eTable 1. Model-Adjusted Means at Each Follow-Up Timepoint eTable 2. Full Model Summary eTable 3. Sensitivity Analysis Using Multiple Imputation and Inverse Probability Weighting eTable 4. Subgroup Analysis for Age: Model-Adjusted Means at Each Follow-Up Timepoint eTable 5. Subgroup Analysis for Symptom Duration: Model-Adjusted Means at Each Follow-Up Timepoint [file jamanetwopen-e2459567-s002.pdf]

Supplementary Online Content

Kim HS, Schauer JM, Kan AK, et al. Emergency department vestibular rehabilitation therapy for dizziness and vertigo: a nonrandomized clinical trial. *JAMA Netw Open*. 2025;8(2):e2459567. doi:10.1001/jamanetworkopen.2024.59567

eFigure 1. Numeric Rating Score (NRS) Over Time

eFigure 2. Global Rating of Change (GROC) Score Over Time

eTable 1. Model-Adjusted Means at Each Follow-Up Timepoint

eTable 2. Full Model Summary

eTable 3. Sensitivity Analysis Using Multiple Imputation and Inverse Probability Weighting

eTable 4. Subgroup Analysis for Age: Model-Adjusted Means at Each Follow-Up Timepoint

eTable 5. Subgroup Analysis for Symptom Duration: Model-Adjusted Means at Each Follow-Up Timepoint

This supplementary material has been provided by the authors to give readers additional information about their work.

© 2025 Kim HS et al. *JAMA Network Open.*

eFigure 1: Numeric Rating Score (NRS) over Time

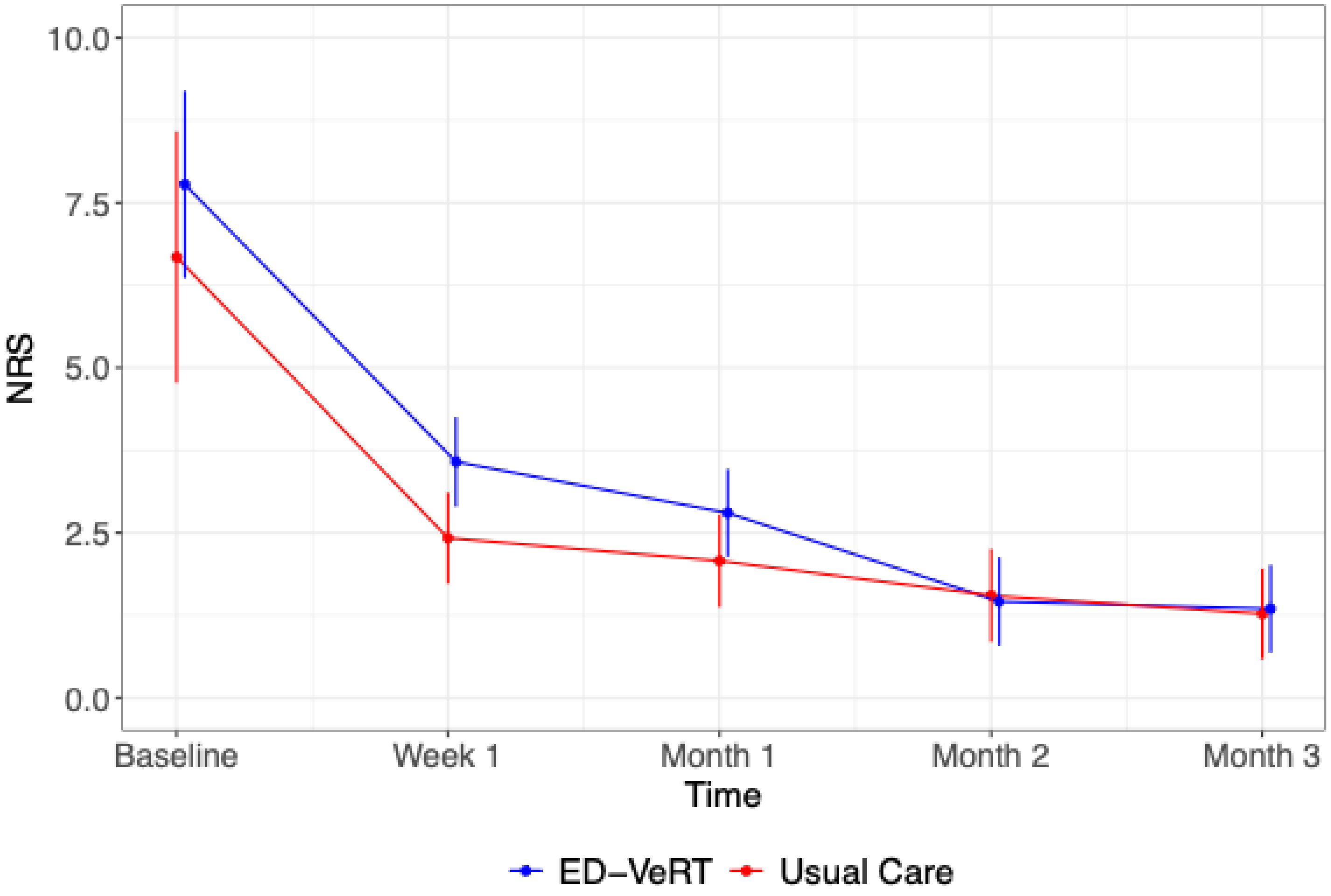

© 2025 Kim HS et al. *JAMA Network Open.*

eFigure 2: Global Rating of Change (GROC) Score Over Time

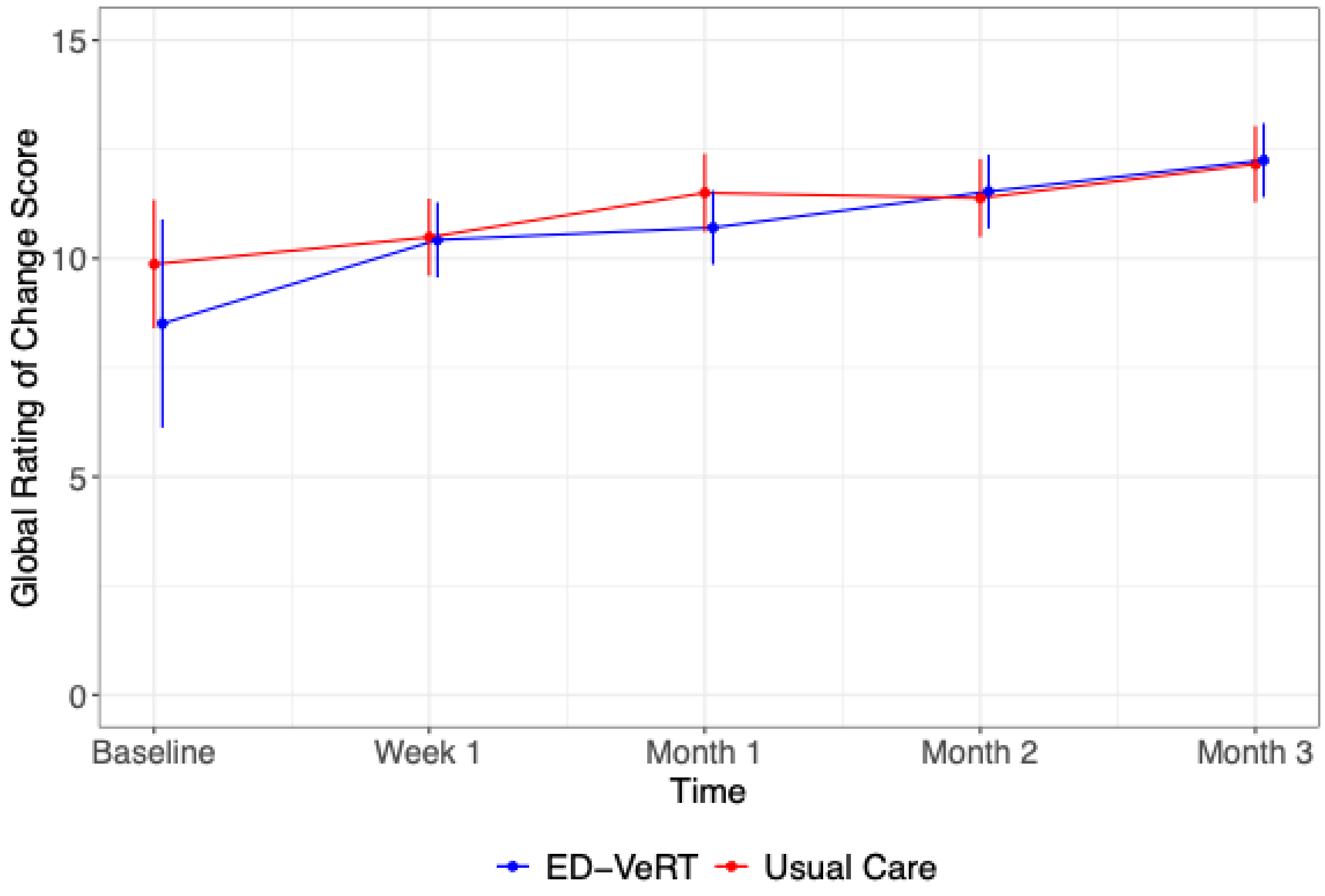

© 2025 Kim HS et al. *JAMA Network Open.*

eTable 1: Model-Adjusted Means at Each Follow-Up Timepoint

		Week 1	Month 1	Month 2	Month 3
DHI	Control	31.56 (3.39) [24.91, 38.21]	26.35 (3.84) [18.81, 33.88]	23.95 (3.54) [17, 30.9]	22.22 (3.5) [15.35, 29.09]
	ED-VeRT	36.5 (3.44) [29.76, 43.25]	30.59 (3.48) [23.76, 37.41]	23.92 (3.51) [17.04, 30.8]	20.54 (3.53) [13.62, 27.46]
	ED-VeRT – Control	4.94 (4.83) [-4.52, 14.4]	4.24 (5.04) [-5.63, 14.12]	-0.02 (5.04) [-9.91, 9.86]	-1.68 (4.89) [-11.27, 7.9]
VAAI-9	Control	22 (2.28) [17.52, 26.47]	18.36 (2.4) [13.65, 23.08]	16.82 (2.42) [12.08, 21.56]	16.33 (2.4) [11.63, 21.04]
	ED-VeRT	24.03 (2.42) [19.28, 28.77]	20.27 (2.27) [15.82, 24.72]	15.66 (2.38) [10.99, 20.33]	14.07 (2.29) [9.57, 18.56]
	ED-VeRT – Control	2.03 (3.31) [-4.45, 8.51]	1.91 (3.29) [-4.54, 8.35]	-1.16 (3.41) [-7.83, 5.52]	-2.27 (3.13) [-8.4, 3.86]
Sedating Medication Use	Control	17% (0.07) [0.02, 0.32]	13% (0.07) [-0.01, 0.27]	13% (0.07) [0, 0.27]	11% (0.06) [-0.01, 0.24]
	ED-VeRT	34% (0.11) [0.12, 0.55]	13% (0.06) [0, 0.25]	7% (0.05) [-0.02, 0.16]	6%(0.04) [-0.01, 0.13]
	ED-VeRT – Control	OR=2.45 (0.18) [0.68, 8.77]	OR=0.97 (0.33) [0.23, 4.04]	OR=0.5 (0.41) [0.1, 2.62]	OR=0.49 (0.4) [0.1, 2.48]
NRS	Control	2.28 (0.34) [1.61, 2.94]	2.05 (0.35) [1.37, 2.73]	1.71 (0.35) [1.03, 2.38]	1.49 (0.35) [0.8, 2.17]
	ED-VeRT	3.01 (0.35) [2.33, 3.69]	2.72 (0.32) [2.09, 3.35]	1.55 (0.34) [0.88, 2.22]	1.46 (0.33) [0.82, 2.11]
	ED-VeRT – Control	0.73 (0.48) [-0.21, 1.67]	0.67 (0.48) [-0.27, 1.62]	-0.16 (0.49) [-1.11, 0.8]	-0.03 (0.48) [-0.96, 0.91]
GROC	Control	10.73 (0.46) [9.82, 11.64]	11.47 (0.45) [10.58, 12.35]	11.18 (0.45) [10.3, 12.07]	11.88 (0.42) [11.05, 12.71]
	ED-VeRT	10.74 (0.43) [9.9, 11.58]	10.62 (0.43) [9.78, 11.47]	11.48 (0.43) [10.64, 12.32]	12.06 (0.42) [11.23, 12.89]
	ED-VeRT – Control	0.01 (0.64) [-1.25, 1.27]	-0.84 (0.61) [-2.04, 0.36]	0.29 (0.61) [-0.91, 1.5]	0.18 (0.6) [-1, 1.35]

For each outcome and timepoint, the table reports model-adjusted (GLMM) mean (SD) and [95%CI].

© 2025 Kim HS et al. *JAMA Network Open.*

eTable 2: Full Model Summary

	DHI	VAAI-9	Sedating Medication Use	NRS	GROC
Intercept	17.378 (8.620) [0.416, 34.340] t=2.016 (0.045)	13.967 (5.964) [2.230, 25.705] t=2.342 (0.020)	−1.762 (0.895) [−3.524, −0.001] t=−1.969 (0.050)	2.010 (1.016) [0.009, 4.010] t=1.977 (0.049)	9.259 (1.091) [7.112, 11.406] t=8.487 (<0.001)
ED-VeRT	8.238 (4.547) [−0.709, 17.186] t=1.812 (0.071)	3.534 (3.229) [−2.820, 9.888] t=1.094 (0.275)	0.895 (0.651) [−0.386, 2.177] t=1.375 (0.170)	1.153 (0.489) [0.191, 2.115] t=2.359 (0.019)	−0.062 (0.627) [−1.295, 1.171] t=−0.099 (0.921)
Month1	−6.569 (2.762) [−12.003, −1.135] t=−2.379 (0.018)	−5.708 (2.161) [−9.961, −1.455] t=−2.642 (0.009)	−0.318 (0.674) [−1.648, 1.012] t=−0.472 (0.638)	−0.347 (0.358) [−1.051, 0.357] t=−0.969 (0.333)	1.016 (0.439) [0.151, 1.881] t=2.312 (0.021)
Month2	−8.436 (2.822) [−13.990, −2.882] t=−2.989 (0.003)	−7.170 (2.206) [−11.512, −2.829] t=−3.250 (0.001)	−0.290 (0.636) [−1.543, 0.963] t=−0.456 (0.649)	−0.871 (0.365) [−1.589, −0.153] t=−2.387 (0.018)	0.897 (0.448) [0.015, 1.779] t=2.002 (0.046)
Month3	−11.573 (2.739) [−16.964, −6.182] t=−4.225 (<0.001)	−8.026 (2.159) [−12.276, −3.776] t=−3.717 (<0.001)	−0.479 (0.664) [−1.788, 0.830] t=−0.721 (0.471)	−1.149 (0.357) [−1.851, −0.447] t=−3.222 (0.001)	1.673 (0.438) [0.810, 2.536] t=3.815 (<0.001)
Baseline Outcome	0.480 (0.082) [0.319, 0.642] t=5.844 (<0.001)	0.410 (0.088) [0.236, 0.583] t=4.646 (<0.001)	0.668 (0.532) [−0.378, 1.715] t=1.257 (0.210)	0.135 (0.093) [−0.048, 0.319] t=1.452 (0.148)	0.185 (0.084) [0.019, 0.350] t=2.195 (0.029)
Age	−0.048 (0.120) [−0.284, 0.188] t=−0.399 (0.690)	−0.027 (0.079) [−0.182, 0.129] t=−0.338 (0.735)	0.002 (0.014) [−0.025, 0.029] t=0.146 (0.884)	−0.009 (0.011) [−0.031, 0.013] t=−0.805 (0.422)	−0.012 (0.015) [−0.042, 0.019] t=−0.752 (0.453)
Male	−7.809 (4.197) [−16.067, 0.449] t=−1.861 (0.064)	−5.671 (2.749) [−11.082, −0.260] t=−2.063 (0.040)	−0.509 (0.501) [−1.494, 0.477] t=−1.016 (0.311)	−0.243 (0.394) [−1.019, 0.532] t=−0.617 (0.537)	0.265 (0.513) [−0.745, 1.275] t=0.517 (0.605)
ED-VERT x Month 1	−2.744 (3.821) [−10.263, 4.775] t=−0.718 (0.473)	−1.479 (2.997) [−7.376, 4.418] t=−0.494 (0.622)	−0.927 (0.885) [−2.672, 0.818] t=−1.047 (0.296)	−0.428 (0.494) [−1.400, 0.544] t=−0.867 (0.387)	−0.734 (0.607) [−1.928, 0.459] t=−1.211 (0.227)
ED-VERT x Month 2	−7.829 (3.873) [−15.451, −0.207] t=−2.021 (0.044)	−5.042 (3.045) [−11.035, 0.951] t=−1.656 (0.099)	−1.585 (0.974) [−3.508, 0.338] t=−1.626 (0.106)	−1.244 (0.501) [−2.231, −0.258] t=−2.482 (0.014)	0.211 (0.614) [−0.998, 1.419] t=0.343 (0.732)
ED-VERT x Month 3	−9.253 (3.812) [−16.754, −1.752] t=−2.428 (0.016)	−5.347 (3.001) [−11.253, 0.559] t=−1.782 (0.076)	−1.608 (0.966) [−3.511, 0.296] t=−1.665 (0.097)	−1.077 (0.494) [−2.048, −0.105] t=−2.180 (0.030)	0.152 (0.607) [−1.042, 1.347] t=0.251 (0.802)

Columns correspond to outcomes, rows to fixed effects. Cells report raw regression coefficients (SE) and [95%CI] along with test statistics (t=) and (p-values).

© 2025 Kim HS et al. *JAMA Network Open.*

eTable 3: Sensitivity Analysis Using Multiple Imputation and Inverse Probability Weighting

	DHI	VAAI-9	Sedating Medication Use	NRS	GROC
Intercept	17.427 (8.568)<br>[0.537, 34.318]<br>t=2.034 (0.043)	12.426 (5.485)<br>[1.629, 23.222]<br>t=2.266 (0.024)	−1.871 (0.969)<br>[−3.779, 0.036]<br>t=−1.932 (0.054)	2.042 (0.850)<br>[0.366, 3.717]<br>t=2.401 (0.017)	9.418 (1.013)<br>[7.419, 11.417]<br>t=9.297 (<0.001)
ED-VeRT	4.942 (4.826)<br>[−4.561, 14.445]<br>t=1.024 (0.307)	2.029 (3.308)<br>[−4.490, 8.549]<br>t=0.613 (0.540)	1.003 (0.669)<br>[−0.314, 2.320]<br>t=1.499 (0.135)	0.732 (0.478)<br>[−0.210, 1.674]<br>t=1.530 (0.127)	0.010 (0.643)<br>[−1.259, 1.279]<br>t=0.015 (0.988)
Month1	−5.215 (4.166)<br>[−13.456, 3.027]<br>t=−1.252 (0.213)	−3.633 (2.659)<br>[−8.878, 1.613]<br>t=−1.366 (0.174)	−0.315 (0.670)<br>[−1.639, 1.008]<br>t=−0.471 (0.638)	−0.231 (0.426)<br>[−1.073, 0.610]<br>t=−0.543 (0.588)	0.735 (0.555)<br>[−0.363, 1.832]<br>t=1.323 (0.188)
Month2	−7.614 (3.973)<br>[−15.458, 0.231]<br>t=−1.917 (0.057)	−5.181 (2.696)<br>[−10.502, 0.140]<br>t=−1.922 (0.056)	−0.223 (0.624)<br>[−1.455, 1.008]<br>t=−0.357 (0.721)	−0.571 (0.414)<br>[−1.387, 0.245]<br>t=−1.379 (0.169)	0.454 (0.534)<br>[−0.600, 1.508]<br>t=0.851 (0.396)
Month3	−9.341 (3.683)<br>[−16.595, −2.087]<br>t=−2.537 (0.012)	−5.662 (2.686)<br>[−10.962, −0.362]<br>t=−2.108 (0.036)	−0.456 (0.666)<br>[−1.770, 0.858]<br>t=−0.685 (0.494)	−0.789 (0.417)<br>[−1.613, 0.034]<br>t=−1.891 (0.060)	1.149 (0.540)<br>[0.083, 2.215]<br>t=2.128 (0.035)
Baseline Outcome	0.415 (0.083)<br>[0.251, 0.580]<br>t=4.989 (<0.001)	0.374 (0.083)<br>[0.210, 0.537]<br>t=4.493 (<0.001)	0.716 (0.586)<br>[−0.437, 1.870]<br>t=1.222 (0.223)	0.113 (0.074)<br>[−0.033, 0.259]<br>t=1.529 (0.128)	0.172 (0.075)<br>[0.025, 0.319]<br>t=2.296 (0.022)
Age	−0.017 (0.117)<br>[−0.248, 0.215]<br>t=−0.140 (0.888)	−0.010 (0.072)<br>[−0.152, 0.132]<br>t=−0.140 (0.889)	0.001 (0.015)<br>[−0.029, 0.031]<br>t=0.060 (0.952)	−0.009 (0.010)<br>[−0.028, 0.010]<br>t=−0.910 (0.364)	−0.007 (0.014)<br>[−0.034, 0.020]<br>t=−0.539 (0.590)
Male	−6.715 (3.657)<br>[−13.907, 0.477]<br>t=−1.836 (0.067)	−4.355 (2.433)<br>[−9.141, 0.432]<br>t=−1.790 (0.074)	−0.589 (0.542)<br>[−1.657, 0.478]<br>t=−1.086 (0.278)	−0.277 (0.328)<br>[−0.922, 0.368]<br>t=−0.846 (0.398)	0.254 (0.444)<br>[−0.619, 1.127]<br>t=0.573 (0.567)
ED-VERT x Month 1	−0.700 (5.596)<br>[−11.755, 10.355]<br>t=−0.125 (0.901)	−0.123 (3.719)<br>[−7.460, 7.215]<br>t=−0.033 (0.974)	−1.056 (0.879)<br>[−2.790, 0.677]<br>t=−1.202 (0.231)	−0.060 (0.601)<br>[−1.247, 1.128]<br>t=−0.099 (0.921)	−0.853 (0.732)<br>[−2.296, 0.590]<br>t=−1.165 (0.245)
ED-VERT x Month 2	−4.967 (5.372)<br>[−15.565, 5.631]<br>t=−0.924 (0.356)	−3.185 (4.037)<br>[−11.172, 4.803]<br>t=−0.789 (0.432)	−1.799 (0.981)<br>[−3.738, 0.139]<br>t=−1.835 (0.069)	−0.888 (0.614)<br>[−2.100, 0.325]<br>t=−1.447 (0.150)	0.285 (0.737)<br>[−1.170, 1.740]<br>t=0.386 (0.700)
ED-VERT x Month 3	−6.625 (5.567)<br>[−17.620, 4.369]<br>t=−1.190 (0.236)	−4.299 (3.628)<br>[−11.451, 2.853]<br>t=−1.185 (0.237)	−1.765 (0.973)<br>[−3.685, 0.155]<br>t=−1.815 (0.071)	−0.758 (0.582)<br>[−1.905, 0.390]<br>t=−1.302 (0.195)	0.167 (0.745)<br>[−1.304, 1.638]<br>t=0.224 (0.823)

Columns correspond to outcomes, rows to fixed effects. Cells report raw regression coefficients (SE) and [95%CI] along with test statistics (t=) and (p-values).

© 2025 Kim HS et al. *JAMA Network Open.*

eTable 4: Subgroup Analysis for Age: Model-Adjusted Means at Each Follow-Up Timepoint

		Week 1	Month 1	Month 2	Month 3
**DHI** Full Cohort	**Control**	31.56 (3.39) [24.91, 38.21]	26.35 (3.84) [18.81, 33.88]	23.95 (3.54) [17, 30.9]	22.22 (3.5) [15.35, 29.09]
	**ED-VeRT**	36.5 (3.44) [29.76, 43.25]	30.59 (3.48) [23.76, 37.41]	23.92 (3.51) [17.04, 30.8]	20.54 (3.53) [13.62, 27.46]
	**ED-VeRT – Control**	4.94 (4.83) [-4.52, 14.4]	4.24 (5.04) [-5.63, 14.12]	-0.02 (5.04) [-9.91, 9.86]	-1.68 (4.89) [-11.27, 7.9]
**DHI** ≤65yo (n=91)	**Control**	31.21 (3.72) [23.93, 38.5]	27.46 (3.84) [19.92, 34.99]	25.02 (3.87) [17.42, 32.61]	21.58 (3.84) [14.05, 29.11]
	**ED-VeRT**	38.65 (3.92) [30.97, 46.34]	27.89 (4.07) [19.91, 35.87]	19.62 (4.06) [11.66, 27.58]	12.59 (4.03) [4.69, 20.5]
	**ED-VeRT – Control**	7.44 (5.42) [-3.18, 18.07]	0.43 (5.62) [-10.58, 11.44]	-5.4 (5.63) [-16.43, 5.63]	-8.99 (5.59) [-19.94, 1.96]
**DHI** >65yo (n=34)	**Control**	43.91 (8.11) [28, 59.81]	28.22 (8.48) [11.6, 44.85]	28.71 (8.37) [12.32, 45.11]	27.05 (8.15) [11.09, 43.02]
	**ED-VeRT**	51.06 (7.4) [36.56, 65.56]	45.05 (7.09) [31.15, 58.95]	39.9 (7.14) [25.9, 53.89]	39.08 (7.14) [25.1, 53.07]
	**ED-VeRT – Control**	7.15 (8.37) [-9.24, 23.55]	16.82 (8.58) [0.01, 33.64]	11.18 (8.45) [-5.38, 27.75]	12.03 (8.2) [-4.05, 28.1]
**VAAI-9** Full Cohort	**Control**	22 (2.28) [17.52, 26.47]	18.36 (2.4) [13.65, 23.08]	16.82 (2.42) [12.08, 21.56]	16.33 (2.4) [11.63, 21.04]
	**ED-VeRT**	24.03 (2.42) [19.28, 28.77]	20.27 (2.27) [15.82, 24.72]	15.66 (2.38) [10.99, 20.33]	14.07 (2.29) [9.57, 18.56]
	**ED-VeRT – Control**	2.03 (3.31) [-4.45, 8.51]	1.91 (3.29) [-4.54, 8.35]	-1.16 (3.41) [-7.83, 5.52]	-2.27 (3.13) [-8.4, 3.86]
**VAAI-9** ≤65yo (n=91)	**Control**	22.35 (2.46) [17.52, 27.18]	16.88 (2.52) [11.93, 21.82]	14.84 (2.58) [9.79, 19.9]	13.7 (2.51) [8.77, 18.63]
	**ED-VeRT**	26.98 (2.59) [21.89, 32.06]	17.11 (2.48) [12.24, 21.98]	13.43 (2.57) [8.4, 18.46]	11.55 (2.51) [6.64, 16.46]
	**ED-VeRT – Control**	4.63 (3.64) [-2.5, 11.76]	0.23 (3.6) [-6.83, 7.3]	-1.42 (3.69) [-8.65, 5.81]	-2.15 (3.6) [-9.2, 4.91]
**VAAI-9** >65yo (n=34)	**Control**	25.15 (5.07) [15.21, 35.1]	18.73 (5.07) [8.78, 28.67]	19.65 (4.78) [10.28, 29.02]	19.17 (4.68) [10, 28.34]
	**ED-VeRT**	30.74 (4.3) [22.31, 39.17]	30.07 (4.35) [21.54, 38.59]	21.51 (4.18) [13.32, 29.7]	22.1 (4.3) [13.67, 30.53]
	**ED-VeRT – Control**	5.59 (6.56) [-7.28, 18.45]	11.34 (6.58) [-1.56, 24.24]	1.86 (6.2) [-10.3, 14.01]	2.93 (6.22) [-9.26, 15.11]

For each outcome and timepoint, the table reports model-adjusted (GLMM) mean (SD) and [95%CI].
The model for Sedating Medication Use did not converge, thus subgroup analyses are not reported

© 2025 Kim HS et al. *JAMA Network Open.*

eTable 5: Subgroup Analysis for Symptom Duration: Model-Adjusted Means at Each Follow-Up Timepoint

		Week 1	Month 1	Month 2	Month 3
**DHI** Full Cohort	**Control**	31.56 (3.39) [24.91, 38.21]	26.35 (3.84) [18.81, 33.88]	23.95 (3.54) [17, 30.9]	22.22 (3.5) [15.35, 29.09]
	**ED-VeRT**	36.5 (3.44) [29.76, 43.25]	30.59 (3.48) [23.76, 37.41]	23.92 (3.51) [17.04, 30.8]	20.54 (3.53) [13.62, 27.46]
	**ED-VeRT – Control**	4.94 (4.83) [-4.52, 14.4]	4.24 (5.04) [-5.63, 14.12]	-0.02 (5.04) [-9.91, 9.86]	-1.68 (4.89) [-11.27, 7.9]
**DHI** ≤ 3 days (n=85)	**Control**	31.21 (3.72) [23.93, 38.5]	27.46 (3.84) [19.92, 34.99]	25.02 (3.87) [17.42, 32.61]	21.58 (3.84) [14.05, 29.11]
	**ED-VeRT**	38.65 (3.92) [30.97, 46.34]	27.89 (4.07) [19.91, 35.87]	19.62 (4.06) [11.66, 27.58]	12.59 (4.03) [4.69, 20.5]
	**ED-VeRT – Control**	7.44 (5.42) [-3.18, 18.07]	0.43 (5.62) [-10.58, 11.44]	-5.4 (5.63) [-16.43, 5.63]	-8.99 (5.59) [-19.94, 1.96]
**DHI** > 3 days (n=40)	**Control**	43.91 (8.11) [28, 59.81]	28.22 (8.48) [11.6, 44.85]	28.71 (8.37) [12.32, 45.11]	27.05 (8.15) [11.09, 43.02]
	**ED-VeRT**	51.06 (7.4) [36.56, 65.56]	45.05 (7.09) [31.15, 58.95]	39.9 (7.14) [25.9, 53.89]	39.08 (7.14) [25.1, 53.07]
	**ED-VeRT – Control**	7.15 (8.37) [-9.24, 23.55]	16.82 (8.58) [0.01, 33.64]	11.18 (8.45) [-5.38, 27.75]	12.03 (8.2) [-4.05, 28.1]
**VAAI-9** Full Cohort	**Control**	22 (2.28) [17.52, 26.47]	18.36 (2.4) [13.65, 23.08]	16.82 (2.42) [12.08, 21.56]	16.33 (2.4) [11.63, 21.04]
	**ED-VeRT**	24.03 (2.42) [19.28, 28.77]	20.27 (2.27) [15.82, 24.72]	15.66 (2.38) [10.99, 20.33]	14.07 (2.29) [9.57, 18.56]
	**ED-VeRT – Control**	2.03 (3.31) [-4.45, 8.51]	1.91 (3.29) [-4.54, 8.35]	-1.16 (3.41) [-7.83, 5.52]	-2.27 (3.13) [-8.4, 3.86]
**VAAI-9** ≤ 3 days (n=85)	**Control**	21.96 (2.64) [16.78, 27.15]	18.24 (2.67) [13.01, 23.47]	16.63 (2.69) [11.36, 21.9]	15.89 (2.66) [10.67, 21.1]
	**ED-VeRT**	26.9 (2.73) [21.55, 32.26]	17.39 (2.79) [11.91, 22.86]	12.16 (2.78) [6.7, 17.61]	8.81 (2.76) [3.4, 14.22]
	**ED-VeRT – Control**	4.94 (3.83) [-2.57, 12.44]	-0.85 (3.89) [-8.48, 6.78]	-4.47 (3.9) [-12.11, 3.16]	-7.08 (3.86) [-14.64, 0.49]
**VAAI-9** > 3 days (n=40)	**Control**	31.76 (5.63) [20.72, 42.79]	19.52 (5.76) [8.22, 30.82]	18.7 (5.64) [7.66, 29.75]	18.13 (5.44) [7.46, 28.8]
	**ED-VeRT**	29.37 (5.03) [19.51, 39.22]	27.13 (4.63) [18.06, 36.21]	22.44 (4.68) [13.26, 31.61]	24.52 (4.67) [15.37, 33.68]
	**ED-VeRT – Control**	-2.39 (6.15) [-14.46, 9.67]	7.61 (6.01) [-4.18, 19.4]	3.73 (5.88) [-7.78, 15.25]	6.39 (5.63) [-4.63, 17.42]

For each outcome and timepoint, the table reports model-adjusted (GLMM) mean (SD) and [95%CI].
The model for Sedating Medication Use did not converge, thus subgroup analyses are not reported

© 2025 Kim HS et al. *JAMA Network Open.*
