# Supplementary material for: Emergency Department Vestibular Rehabilitation Therapy for Dizziness and Vertigo: A Nonrandomized Clinical Trial
Source: JAMA Netw Open. 2025 Feb 14;8(2):e2459567. doi: 10.1001/jamanetworkopen.2024.59567 (PMC11829232; doi:10.1001/jamanetworkopen.2024.59567)
Supplement: Supplement 3. — Data Sharing Statement [file jamanetwopen-e2459567-s003.pdf]

Data Sharing Statement

Kim. Emergency Department Vestibular Rehabilitation Therapy for Dizziness and Vertigo.
JAMA Netw Open. Published February 14, 2025. doi:10.1001/jamanetworkopen.2024.59567

Data
Additional Information: NCT05122663
Data available: Yes
Data types: Deidentified participant data, Data dictionary
How to access data: Upon reasonable request and completion of appropriate data use authorization agreement.
When available: With publication

Supporting Documents
Document types: None

Additional Information
Who can access the data: Not applicable
Types of analyses: N/A
Mechanisms of data availability: N/A
Any additional restrictions: N/A
